# Risk of dementia and cognitive dysfunction in individuals with diabetes or elevated blood glucose

**DOI:** 10.1017/S2045796019000374

**Published:** 2019-08-28

**Authors:** I K Wium-Andersen, J Rungby, M B Jørgensen, A Sandbæk, M Osler, M K Wium-Andersen

**Affiliations:** 1 Psychiatric Center Copenhagen, Department O, Copenhagen, Denmark; 2 Center for Clinical Research and Disease Prevention, Frederiksberg Hospital, Denmark; 3 Departments of Endocrinology, Bispebjerg-Frederiksberg Hospital and Aarhus University Hospital, Denmark; 4 Department of Public Health, Section for General Practices, University of Aarhus, Denmark; 5 Section for Epidemiology, Department of Public Health, University of Copenhagen, Denmark

**Keywords:** Cognition, dementia, diabetes, dysglycemia, HbA1c

## Abstract

**Aims:**

To determine the risk of dementia in patients with type 1 or type 2 diabetes and in individuals with glycosylated haemoglobin, type A1C (HbA1c) of ⩾48 mmol/mol, which is the diagnostic limit for diabetes.

**Methods:**

We included the following cohorts: all incident diabetes cases aged 15 or above registered in the National Diabetes Registry (NDR) from January 2000 through December 2012 (*n* = 148 036) and a reference population, adult participants from the Glostrup cohort (*n* = 16 801), the ADDITION Study (*n* = 26 586) and Copenhagen Aging and Midlife Biobank (CAMB) (*n* = 5408). Using these cohorts, we analysed if a diagnosis of type 1 or type 2 diabetes in the NDR or HbA1c level of ⩾ 6.5% (48 mmol/mol) in the cohorts increased risk of dementia in the Danish National Patient Registry or cognitive performance assessed by the Intelligenz-Struktur-Test 2000R (IST2000R).

**Results:**

A diagnosis of type 1 or type 2 diabetes in the NDR was associated with increased risk of dementia diagnosed both before or after age 65 as well as across different subtypes of dementia. Self-reported diabetes or high HbA1c levels were associated with lower cognitive performance (*p* = 0.004), while high HbA1c was associated with increased risk of dementia (HR 1.94 (1.10–3.44) in the Glostrup cohort but not in the ADDITION Study (HR 0.96 (0.57–1.61)).

**Conclusions:**

Both type 1 and type 2 diabetes are associated with an increased risk of dementia, while the importance of screening-detected elevated HbA1c remains less clear.

## Introduction

Dementia is a broad category of diseases characterised by an irreversible decline of cognitive function. Alzheimer's disease is the most common cause of dementia, followed by vascular dementia. Dementia is a leading cause of disability, and with an increasing elderly population worldwide the prevalence of dementia is expected to increase (Umegaki, 2014). Lack of effective treatment calls for the identification of modifiable risk factors and strategies for prevention. Type 2 diabetes has been associated with increased risk of developing all types of dementia, but results have been conflicting and the association between diabetes and dementia is still disputed.

So far, several meta-analyses have reported that diabetes increases risk of Alzheimer's Disease 1.5 times and vascular dementia 2.5 times (Cheng *et al*., 2012; Chatterjee *et al*., 2016; Zhang *et al*., 2017). However, many of the included studies were limited by small sample sizes with an insufficient number of outcomes or limiting controlling for clinical variables such as cholesterol, body mass index (BMI), smoking status, or psychiatric comorbidity. Furthermore, some studies even suggest that type 2 diabetes may not be a risk factor for Alzheimer's disease, or that it may only be a risk factor in a subgroup of patients (Heitner and Dickson, 1997; Hassing *et al*., 2002; Smolina *et al*., 2015; Sherzai *et al*., 2016). Importantly, most previous studies did not specifically characterise participants as type 1 or type 2 diabetes. Only two studies have analysed risk of dementia in type 1 diabetes. In the first study, the overall risk of dementia was increased with a relative risk of 1.65 (95% confidence interval (CI) 1.61–1.68), however the risk of Alzheimer's disease was lower (relative risk 1.10 (95% CI 1.05–1.15)) while the risk of vascular dementia was higher (relative risk 2.21 (95% CI 2.13–2.28)) (Smolina *et al*., 2015). The second study found a threefold increased risk of dementia (Kuo *et al*., 2018), but the study was limited by few endpoints (*n*  =  44 of 1077 type 1 diabetes patients) and did not distinguish between dementia types.

Both hormonal and vascular mechanisms are suggested as common pathological pathways between dementia and diabetes (Nielsen *et al*., 2017; Arnold *et al*., 2018). Under normal circumstances, insulin serves many roles in the brain such as stimulating the growth of neuronal cells and protection of apoptosis and oxidative stress. Peripheral insulin anomalies are thought to cause a decrease in brain insulin levels, disrupting these functions (Blazquez *et al*., 2014). Hyperglycemia in itself is also shown to have a toxic effect on neurons due to the production of advanced glycation end products which cause oxidative damage and neuronal injury (Umegaki, 2014). A few studies have examined the association between glucose levels and dementia and a cohort study from the USA of 2961 men and women followed for 6.7 years suggested that higher glucose levels may be a risk factor for dementia, even among persons without diabetes (Crane *et al*., 2013).

Against this background, the aim of this study was to examine if type 1 diabetes, type 2 diabetes, and dysglycemia were associated with lower cognitive performance and increased risk of dementia. In nationwide registers and population-based cohorts, we tested the following hypotheses:

1. Type 1 and type 2 diabetes are associated with increased risk of dementia after controlling for sociodemographic factors and somatic and psychiatric comorbidity.
2. HbA1c of 48 mmol/mol (6.5%) (which is the limit for diabetes) is associated with increased risk of dementia after controlling for lifestyle-related covariables.
3. Individuals with HbA1c of 48 mmol/mol (6.5%) and/or self-reported diabetes have lower cognitive performance than individuals with HbA1c of 48 mmol/mol (6.5%) and no self-reported diabetes after controlling for lifestyle-related covariables and inflammatory markers.

## Material and methods

### Study population

We used information from four cohorts: A nationwide, register-based cohort with all diabetes cases registered in the National Diabetes Register (NDR) and a matched reference population (Carstensen *et al*., 2011), the Glostrup cohorts (Osler *et al*., 2011), the ADDITION cohort (Lauritzen *et al*., 2000), and the Copenhagen Aging and Midlife Biobank (CAMB) (Avlund *et al*., 2014). An overview of the four cohorts is given in Table 1.

**Table 1.** Baseline characteristics of the National Diabetes Register cohort

	Reference	DM1	DM2
Number (*n*)	144 807	20 261	125 520
*Basic variables*			
Age in years, mean (range)	60.2 (15–104)	52.4 (15–102)	61.7 (15–104)
Men, *n* (%)	81 333 (56)	10 887 (54)	70 806 (56)
Education, *n* (%)			
Basic education	51 839 (36)	8160 (40)	53 733 (43)
Medium education	57 367 (40)	7599 (38)	47 547 (38)
Long education	24 629 (17)	2386 (12)	13 752 (11)
Missing	10 972 (8)	2116 (10)	10 488 (8)
Marital status, *n* (%)			
Married	81 718 (56)	8777 (43)	70 340 (56)
Unmarried	21 910 (15)	6364 (31)	17 405 (14)
Divorced	21 075 (15)	2346 (12)	18 699 (15)
Widow/widower	19 296 (13)	2364 (12)	18 411 (15)
Missing	808 (0.6)	410 (2)	665 (0.5)
*Illness variables*			
Ischemic heart disease, *n* (%)	8327 (6)	2591 (13)	18 237 (15)
Cerebrovascular disease, *n* (%)	5000 (3)	1930 (10)	8463 (7)
Cardiovascular medication, *n* (%)	26 980 (19)	5049 (25)	42 374 (34)
Hypertension, *n* (%)	48 221 (33)	9653 (48)	78 066 (62)
Obesity, *n* (%)	5376 (4)	2124 (10)	18 658 (15)
Hypercholesterolemia, *n* (%)	17 272 (12)	3785 (19)	39 365 (31)
Infection, *n* (%)	18 692 (13)	5672 (28)	28 725 (23)
Chronic obstructive pulmonary disease, *n* (%)	15 344 (11)	2985 (15)	20 992 (17)
Inflammatory disease, *n* (%)	4416 (3)	1258 (6)	7119 (6)
Alcohol use disorder, *n* (%)	2784 (2)	1313 (6)	4505 (4)
Major depression, *n* (%)	23 226 (16)	4034 (20)	26 648 (21)

DM1, Diabetes Mellitus type 1; DM2, Diabetes Mellitus type 2.

Based on patients with diabetes from the National Diabetes Register and a non-diabetic reference group from the Civil Registration System. Individuals with previous dementia are excluded.

The nationwide, register-based cohort was based on information on all patients aged 15 or above registered with incident type 1 or type 2 diabetes in NDR between 1 January 2000 and 31 December 2012. The NDR is based on a validated algorithm which combines information on diabetes ascertained by physician's diagnosis (International Classification of Disease 10 (ICD10) codes DE10–14, DH36.0, DO24) in the Danish National Patients Registry, prescriptions of antidiabetic medication in the Danish Prescription Register, and measures of blood glucose or referrals to feet therapists from the National Health Insurance Service Register (Carstensen *et al*., 2011). A reference population was established by 1:1 matching on gender, age, and municipality on time of diabetes diagnosis using information from the Civil Registration System (Pedersen, 2011). If diabetes type (type 1 diabetes or type 2 diabetes) was unclear (*n*  =  10 303), use of oral antidiabetic medication and/or age at diagnosis >35 was used to define type 2 diabetes. In total 127 369 persons with type 2 diabetes and 20 664 patients with type 1 diabetes and similar numbers in the reference populations were included. A flow chart of individuals included is shown in Supplementary Fig. 1.

In this study, the Glostrup cohort consisted of a pooling of the Inter99, Health2006 and the Danish study of Functional Disorders (DanFunD). Inter99 was a population-based primary intervention study and included 6784 randomly sampled individuals aged 30–60 years who participated (participation rate: 52.5%) in a health examination between 1999 and 2001 (Jorgensen *et al*., 2003). The Health2006 cohort included 3471 randomly sampled individuals, aged 18–69 years, who were examined (participation rate: 44.7%) between 2006 and 2009 (Thuesen *et al*., 2014). DanFunD included 6837 randomly selected individuals, aged 18–72 years (participation rate: 29.5%) who were examined between 2012 and 2014 (Dantoft *et al*., 2017). A total of 16 801 cohort members had measurements of HbA1c. Of these, 21 had dementia before study entry leaving 16 780 for analysis.

The ADDITION study (The Anglo-Danish-Dutch Study of Intensive Treatment in People with Screen Detected Diabetes in Primary Care) was initiated in 1999 and included individuals at risk of diabetes but without a diagnosis of diabetes. In ADDITION-Denmark a total of 163 189 men and women aged 40–69 years received a risk score questionnaire and of these 28 035 with a risk score of ⩾5 visited their family doctor and were included in ADDITION (Lauritzen *et al*., 2011). A total of 26 586 individuals had measurements of HbA1c and as 50 had dementia before study entry, this left us 26 536 individuals available for analysis.

Finally, CAMB was based on three cohorts: the Metropolit study, the Danish Longitudinal Study of Work, Unemployment and Health cohort, and the Copenhagen Perinatal Birth Cohort (Avlund *et al*., 2014). A total of 5575 participants aged 49–53 years were assessed with a test of cognitive performance and blood sampling in a midlife follow-up from 2009 to 2011 with a response rate of 30%. After exclusion of 167 individuals without information on either cognitive performance, self-reported diabetes or HbA1c, 5408 individuals were left for analysis.

### Exposures

In the nationwide register-based study, diabetes was defined by the algorithm described above. In the three other cohorts, we defined diabetes as having baseline HbA1c of 48 mmol/mol (6.5%). In CAMB, diabetes was also defined by self-reported diabetes.

### HbA1c measurements

In Inter99 Glycosylated Haemoglobin, type A1C (HbA1c) was assayed by ion-exchange high-performance liquid chromatography technique (HPLC) (Bio-Rad variant). In Health2006, HbA1c was measured using HPLC on a Tosoh G8 Analyzer. In DanFunD, HbA1c was measured using the VITROS Chemistry Products d%A1c Reagent Kit utilising a quantitative turbidimetric inhibition immunoassay until July 2013 and from July 2013 using the HPLC technique on a Tosoh G8 Analyzer. Levels of HbA1c were similar in all three cohorts. In the ADDITION study, HbA1c was similarly measured using ion-exchange HPLC (Tosoh Bioscience, Redditch, UK) and in the CAMB cohort using ion-exchange HPLC on a Waters 625 LC system together with a Waters photo-diode-array detector model 996 and WISP 717 autosampler for automatic injection of the samples.

All measurements of HbA1c were DCCT (Diabetes Control and Complications Trial) aligned but were converted to IFCC (International Federation of Clinical Chemistry) units before analysis.

### Outcomes

Dementia was ascertained by physician's diagnosis in the Danish National Patient Registry using ICD10 codes F00-F03 and G30–31 (Supplementary Table 1). In the register-based cohort, dementia was subdivided into Alzheimer's dementia, vascular dementia, or other dementia based on ICD10 codes shown in Supplementary Table 1. The validity of dementia diagnoses obtained from this register has been assessed in two independent studies of patients admitted in 2003 (Phung *et al*., 2007), and 2008 (Thygesen *et al*., 2011; Salem *et al*., 2012). They revealed that for diagnosis defined by ICD-10, 70% (Salem *et al*., 2012) and 83% (Phung *et al*., 2007) of dementia cases diagnosed by an external rater confirmed the diagnosis recorded in the register (Thygesen *et al*., 2011) with the lowest validity in younger populations and for vascular dementia.

In CAMB, we used cognitive performance assessed by the Intelligenz-Struktur-Test 2000R (IST2000R) translated into Danish by Hogrefe Publishers (Amthauer, 2000). Three subtests of the IST2000R are included in CAMB: Sentence completion, verbal analogies and number series (Avlund *et al*., 2014).

### Covariables

We included a number of covariables assumed to be associated with diabetes and the risk of dementia. Covariables are listed in Supplementary Table 2. In the nationwide register-based cohort, the Glostrup cohorts, and in ADDITION, education was categorised as basic education (7–9 grade of obligatory schooling), medium education (high school degree/vocational), higher education (more than high school degree) or missing based on data from the Integrated Database for Labor Market Research. In CAMB, education was based on self-reported education and categorised into basic education, skilled worker, short/medium education, long education or other education. Marital status was based on data from the Danish Civil Registration System and categorised as married, unmarried, divorced, or widow/widower. In CAMB, we only had information on whether the participant lived alone or not. Somatic and psychiatric comorbidity in the register-based cohort, the Glostrup cohorts and ADDITION were based on either hospitalisation and/or medication registered in the National Patient Registry and the Danish Prescription Register 5 years preceding inclusion of the study. The ICD10 and ATC codes are shown in Supplementary Table 1. All clinical variables and blood tests were measured at the first visit. In the three population-based cohorts, each participant underwent a physical examination by trained staff following standard operation procedures. BMI (kg/m^2^) was defined as weight in kilograms divided by height in meters squared. In CAMB body fat was measured using a Tanita Multi-Frequency Body Composition Analyzer MC-180MA after standard instructions from the manufacturer. In CAMB, C-reactive protein (CRP) was measured using a high sensitive assay using latex-enhanced turbidimetry (Roche/Hitachi), using assays Tina Quant, Roche Diagnostics GmbH, Mannheim, Germany. Interleukin (IL) 6 and IL-10 were measured by an electro-chemiluminescence multiplex system on Sector 2400 Imager from Meso Scale Discovery (Gaithersburg, USA).

### Statistical analyses

Stata version 14.2 (StataCorp, College Station, TX) was used for statistical analysis. Missing data (<5% for each variable), assumed to be missing at random, were imputed using age, sex, and marital status. More information on missing data is provided in Supplementary Information.

Hypothesis 1 was tested using Cox proportional hazard regression models with age as the underlying time scale. Study entry was the date of inclusion in the NDR or the corresponding date for the reference population. End of follow-up was time of dementia, emigration, or death in the Civil Registration System, or end of follow-up (11 May 2018), whichever came first. Individuals (*n*  =  5433) with a diagnosis of dementia or the use of dementia medication before study entry were excluded before analysis. Due to the interaction of age at dementia diagnosis, the results were stratified into early dementia (<65 years of age) and late dementia (⩾65 years of age). In analyses of early dementia, follow-up was ended at age 65 if individuals were not censored before. In the analyses of late dementia, study entry was at age 65 or later (if the inclusion date into the NDR was after age 65), and consequently, individuals who were younger than 65 years old during follow-up were not included. The proportional hazards assumption was tested graphically by plotting –log(-log(survival)) *v*. log(follow-up time) and no major violations were found after stratification on age at diagnosis. Potential interaction between diabetes and sex on risk of dementia was tested for by introducing a multiplicative interaction term (diabetes × sex) into the model and comparing the models using a likelihood ratio test. Covariables were adjusted for as shown in Table 1 and Supplementary Table 2. Diabetes might be diagnosed during testing for dementia, to account for detection bias we delayed follow-up for 1 year using the *stsplit* option in STATA to divide the follow-up time into 0–1 year and above as done in previous studies (Katon *et al*., 2015). We further examined dementia subtypes. In these analyses, all individuals with any dementia diagnosis or medication before study entry were excluded. If individuals had more dementia diagnoses of different subtypes at different times, we followed individuals until the first dementia diagnosis regardless of which type it was, i.e. only allowing individuals to have one dementia diagnosis. A total of 804 individuals had a diagnosis of Alzheimer's dementia and ‘other dementia’ at the same time and were classified as having Alzheimer's dementia. However, as well as examining only the first subtype diagnosis which may be less valid (e.g. unspecified dementia), in supplementary analyses we also made subtype analyses in which we followed individuals until only the specific dementia subtype, thus allowing individuals to have several different dementia subtypes.

For the second hypothesis, the Glostrup cohorts and ADDITION were analysed separately using a Cox model as described above. Study entry was the day of study participation. A total of 21 individuals in the Glostrup cohorts and 50 individuals in ADDITION diagnosed with dementia or a purchase of dementia medication before study entry were excluded from the analyses. When the proportional hazards assumption was tested no violations were found. Due to a limited number of outcomes, multivariable adjustment was limited to adjustment for age, sex, education, smoking status, physical activity, BMI and depression.

Finally, the third hypothesis was tested in CAMB using multiple regression models to estimate the correlation between HbA1c level and cognitive performance with adjustment as shown in Supplementary Table 2.

## Results

### Risk of dementia in patients with type 1 diabetes or type 2 diabetes

Baseline characteristics of the 20 261 individuals with type 1 diabetes and 125 520 individuals with type 2 diabetes and their respective reference population in the nationwide cohort are shown in Table 1. Median follow-up was 11.3 years (range 0–18 years) for analyses of early dementia (before age 65) and 9.9 years for analyses of late dementia (after age 65). The absolute rate of early dementia was quite low. A total of 88 (0.6%) individuals with type 1 diabetes and 609 (0.8%) with type 2 diabetes were diagnosed with early dementia compared to 344 (0.4%) in the reference population, while the corresponding numbers for late dementia were 597 (6.4%), 5989 (6.8%) and 5913 (6.0%).

**Fig. 1.**
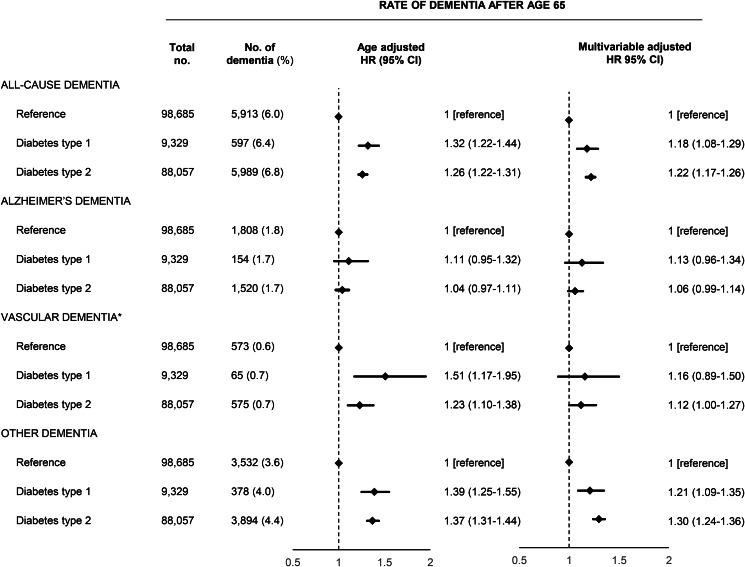
Prospective associations between diabetes type 1 and 2 and dementia above age 65 in the nationwide study. Multivariable adjustment was for age, sex, marital status, education, register-based ischemic heart disease, cerebrovascular disease, vascular disease medication, hypertension, obesity, hypercholesterolemia, infections, chronic obstructive pulmonary disease, inflammatory diseases, depression and alcohol use disorders. HR, hazard ratio; CI, confidence interval.

Type 1 diabetes and type 2 diabetes were associated with increased risk of both early and late dementia. For late dementia, the rates among patients with type 1 and type 2 diabetes were slightly lower for Alzheimer's dementia while slightly higher for vascular dementia and other dementia and for all subtypes, rates had slightly wider confidence intervals for type 1 diabetes due to the lower number of outcomes (Fig. 1). Type 1 diabetes was associated with a HR of 1.18 (1.08–1.29) for all-cause dementia after multivariable adjustment while the corresponding HR for type 2 diabetes was 1.22 (1.17–1.26). In the subtype analyses which allowed for several subtypes of dementia, the estimates were similar but with more narrow confidence intervals due to the slightly higher number of endpoints (Supplementary Fig. 2). We found no interaction with sex (data not shown). The corresponding HRs for early depression before age 65 were slightly higher and is shown in Supplementary Table 3 and 4. The rates of dementia within the first year or after the first year after a diabetes diagnosis did not suggest the presence of detection bias (Supplementary Table 5). When we examined dementia subtypes, the analyses of early Alzheimer's dementia and early vascular dementia were hampered by an insufficient number of dementia outcomes.

### HbA1c levels and risk of dementia

**Fig. 2.**
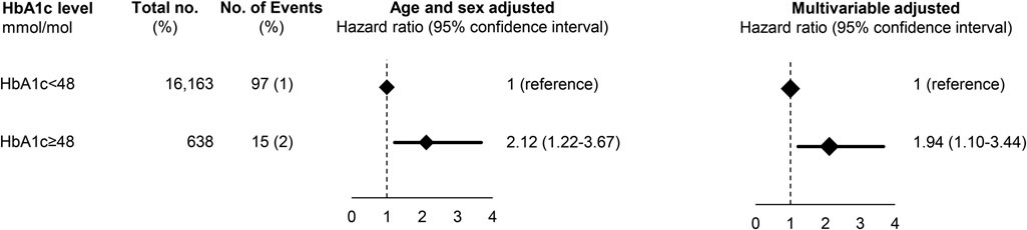
Prospective association between HbA1c and dementia in the combined Glostrup cohorts. Based on 16 780 individuals from the combined Glostrup cohorts. A total of 21 individuals with dementia before study entry were excluded before analysis. Multivariable adjustment was for age, sex, cohort, education, marital status, smoking status, physical activity, body mass index, systolic blood pressure, total cholesterol and register-based ischemic heart disease, cerebrovascular disease and statin use.

**Fig. 3.**
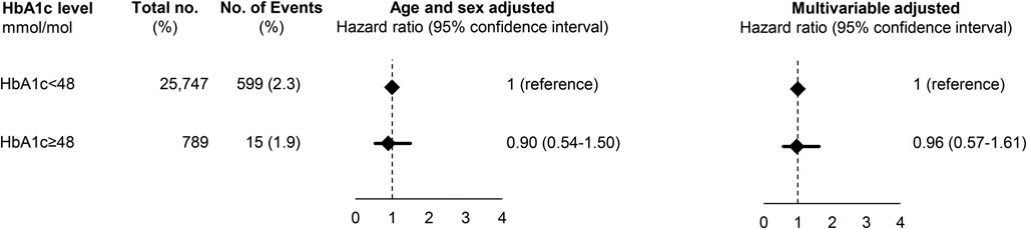
Prospective association between HbA1c and dementia in the Addition Study. Based on 26 536 individuals from the Addition study. A total of 50 individuals with dementia before study entry were excluded before analysis. Multivariable adjustment was for age, sex, education, marital status, smoking status, physical activity, body mass index, systolic blood pressure, and register-based ischemic heart disease, cerebrovascular disease and depression. HR, hazard ratio; CI, confidence interval.

After exclusion of individuals with a previous depression (*N*  =  21 in the Glostrup cohorts and *N*  =  50 in ADDITION), a total of 3.8% (*n*  =  638) had a HbA1c of 48 mmol/mol (corresponds to HbA1c of 6.5%) in the Glostrup cohorts, while in ADDITION, the corresponding proportion was 3.0% (*n*  =  789). Baseline characteristics are shown in Supplementary Table 6 and 7. During a median follow-up of 9.8 years (range 0.01–36) in the Glostrup cohorts and of 12.7 years (range 0.2–14.8) in ADDITION, 112 and 614 developed dementia. In the Glostrup cohorts, HbA1c of 48 mmol/mol was associated with an increased risk of dementia with a HR_age and sex adjusted_ of 2.12 (95% CI 1.22–3.67) which was attenuated to 1.94 (1.10–3.44) after multivariable adjustment for age, sex, education, physical activity, BMI and depression (Fig. 2). In ADDITION, HbA1c was not associated with dementia before or after multivariable adjustment (Fig. 3).

### HbA1c levels and cognitive performance

**Fig. 4.**
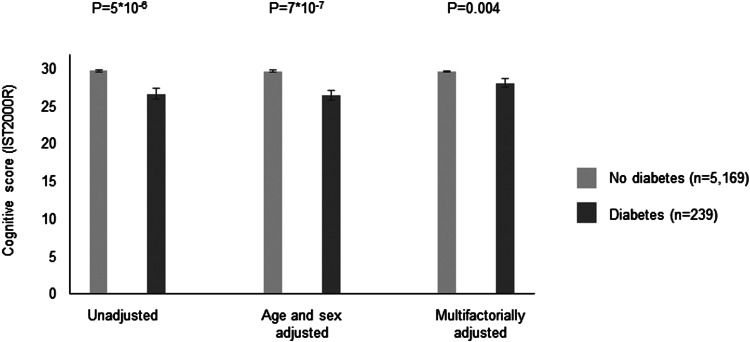
Cross-sectional association between diabetes and cognitive performance on the IST2000R in the CAMB cohort. Based on 5408 individuals in the Copenhagen Aging and Midlife Biobank (CAMB). Diabetes was based on self-reports or a HbA1c ⩾ 48 mmol/mol. IST2000R  =  Intelligenz-Struktur-Test 2000R. Multifactorially adjustment was for age, sex, education, living alone, alcohol, smoking, high sensitive C-reactive protein, interleukin 6, interleukin 10 and body fat percentage.

In the CAMB cohort, 239 (4%) was defined as having diabetes based on self-reports or HbA1c of 48 mmol/mol (6.5%). Characteristics are shown in Supplementary Table 8. Individuals with diabetes had lower cognitive performance scores as compared to individuals without diabetes (*p*  =  5 × 10^−6^) and the difference remained significant after multivariable adjustment (*p*  =  0.004) (Fig. 4).

## Discussion

Our nationwide register-based cohort based on all patients diagnosed with diabetes in Denmark between 2000 and 2012 showed that both type 1 diabetes and type 2 diabetes patients had increased risk of any dementia. The highest risk (up to a 30% increase) was for dementia diagnosed before age 65. In patients with type 1 diabetes, we found no increase in the risk of Alzheimer's Disease or vascular dementia, while patients with type 2 diabetes had increased risk of both vascular dementia and Alzheimer's Disease. Further, in the population-based cohorts HbA1c-defined diabetes was associated with lower cognitive performance, and in one cohort with increased dementia risk, but not in the other.

Previous studies and meta-analyses on the association between type 2 diabetes and dementia have reported much stronger associations, but their results have mainly been driven by two large cohorts from Taiwan (Wang *et al*., 2012) and South Korea (Kimm *et al*., 2011). The strength of an association depends on the distribution of other component causes in the population (Rothman, 1976), and Asian populations may have a different risk profile compared to ours (Zhang *et al*., 2017). Our findings are in line with a Canadian study showing a 16% increased risk of dementia in 225 045 seniors with diabetes (Haroon *et al*., 2015). Also, a previous Danish study of 223 174 diabetes patients examining the combined risk of diabetes and depression on dementia, found that diabetes alone increased risk of dementia 10% after multiple adjustments (Katon *et al*., 2015). However, none of the studies categorised diabetes patients into type 1 or 2.

Our findings for HbA1c were conflicting. In the Glostrup cohorts, HbA1c of 48 mmol/mol (6.5%) increased risk of dementia, but in ADDITION no association between HbA1c and dementia was found. Two previous studies found that HbA1c of 53 mmol/mol (7%) or more was associated with increased risk of dementia, while a diagnosis of diabetes or self-reported diabetes was not associated with dementia (Gao *et al*., 2008; Ramirez *et al*., 2015). However, the studies only had 58 and 67 dementia cases, respectively. A larger cohort study of 232 patients with diabetes and 1835 patients without diabetes found a relationship between sustained elevated glucose levels and dementia risk that was independent of diabetes diagnosis (Crane *et al*., 2013). One possible explanation for the lack of association between HbA1c and dementia in ADDITION could be that study participants were a part of an intervention study receiving intensive care including educational meetings with family physicians and nurses to discuss treatment targets, and lifestyle advice, and more intensive treatment of risk factors such as blood pressure and cholesterol or routine care. After 5 years of follow up, the intensive group had slightly but significantly lower HbA1c, cholesterol and blood pressure (Griffin *et al*., 2011). Participants in Inter99 (*n*  =  6774) in the Glostrup cohorts were also enrolled in an intervention and received lifestyle counselling on smoking cessation or physical activity/diet over 6 months or referral to their general practitioner. However, in Inter99, after 10 years the groups had an equal risk of ischaemic heart disease, stroke, and mortality (Jorgensen *et al*., 2014). Unfortunately, there was no follow-up on HbA1c or other clinical variables. Therefore, the results in ADDITION could suggest that tight metabolic control lowers the risk of dementia in patients with diabetes. However, until now other studies investigating this have failed to find intensive glycemic control to be associated with a lower risk of dementia (Advance Management Committee, 2001) or better cognitive outcome (Areosa Sastre *et al*., 2017).

Finally, we found that self-reported diabetes and/or HbA1c of 48 mmol/mol (6.5%) or above was associated with lower cognitive performance on the IST2000R test after controlling for markers of inflammation. This is in line with previous studies analysing HbA1c levels and cognitive dysfunction (Cukierman-Yaffe *et al*., 2009; Avadhani *et al*., 2015). Inflammation may influence cognition and two studies even found that metabolic syndrome was associated with cognitive dysfunction only in patients with high inflammation (Yaffe *et al*., 2004; Dik *et al*., 2007).

Strengths and limitations: We tested dementia risk in both a large nationwide register-based cohort and two independent cohorts with information on several covariables including education and comorbidity and additionally clinical covariables in the population-based cohorts including lifestyle factors, BMI, blood pressure and cholesterol level. It may limit our results, especially in the Glostrup cohorts that participation rates were relatively low. However, the nationwide register-based cohort covers all individuals diagnosed with diabetes and we had a nearly complete follow-up for hospital diagnosis and prescription records which reduces loss to follow-up and any associated bias. Furthermore, despite a large sample size in the Glostrup, ADDITION and CAMB cohorts, numbers of endpoints were relatively small and consequently, we lack power to explore repeated measures of HbA1c. However, in the nationwide study, we had a relatively large number of endpoints due to the large number of individuals included. Unfortunately, we did not have clinical covariables such as BMI, blood pressure, cholesterol level or inflammatory markers available in this study, which we had in the population-based cohorts. We did not include antidiabetic medication in our analysis since this was not regarded as confounders but potential mediators of the association. Median follow-up time in all cohorts was relatively short (9.8–12.7 years), limiting our results as mean age at end of follow-up ranged from 56 to 57 years in type 1 diabetes and their reference population and 71–72 years in type 2 diabetes and their reference population. In CAMB, we only had one measurement of cognitive performance and could not evaluate changes over time. Also, we only had three subtests of IST2000R which were primarily testing crystalised verbal cognitive ability, which may not be a sensitive measure of midlife cognitive changes (Avlund *et al*., 2014). Overall, the validity of dementia in the National Patient Register is high, however, Alzheimer's Disease may be underdiagnosed, and vascular dementia has substantially lower validity (Phung *et al*., 2007). We found a high proportion of dementia classified as other dementia. More than 91% of the dementia cases in this category was diagnosed ‘unspecified dementia’ (DF03 and G31.9) (data not shown) while the rest was Lewy-body dementia and fronto-temporal dementia. A validation study of dementia diagnosis in the Danish registers showed that a substantial number of cases registered as unspecified dementia is, in reality, Alzheimer's Disease (Phung *et al*., 2007). In our study, these cases may have provided the extra power to detect a significantly increased risk of Alzheimer's Disease in type 1 diabetes or further accentuate the risk of Alzheimer's Disease in type 2 diabetes.

Based on nationwide data on a large diabetes population and previous evidence, we conclude that both type 1 diabetes and type 2 diabetes are associated with a higher risk of dementia and a lower cognitive performance, while the importance of single elevated HbA1c measure could be questioned.

## Supplementary material

For supplementary material accompanying this paper visit http://dx.doi.org/10.1017/S2045796019000374. click here to view supplementary material

## Availability of data and materials

Due to the new general data protection regulation, data cannot be shared.
