# Supplementary material for: Risk of dementia and cognitive dysfunction in individuals with diabetes or elevated blood glucose
Source: Epidemiol Psychiatr Sci. 2019 Aug 28;29:e43. doi: 10.1017/S2045796019000374 (PMC8061228; doi:10.1017/S2045796019000374)
Supplement: Supplementary file 1 [file S2045796019000374sup001.docx]

**Supplementary Information**

Risk of dementia and cognitive dysfunction in individuals with diabetes or elevated blood glucose in four large cohorts

I K Wium-Andersen, J Rungby, M B Jørgensen, A Sandbæk, M Osler, M K Wium-Andersen

[Supplementary text 3](#_Toc10708871)

[Missing information in the cohorts 3](#_Toc10708872)

[Supplementary Tables and Figures 4](#_Toc10708873)

[Supplementary Table 1. ICD10 and ATC codes used to define endpoints and covariables in the cohorts. 4](#_Toc10708874)

[Supplementary Table 2. Exposure, outcomes, and covariables in the four cohorts. 5](#_Toc10708875)

[Supplementary Table 3. Prospective associations between diabetes type 1 and type 2 and dementia subtypes before age 65. 6](#_Toc10708876)

[Supplementary Table 4. Prospective associations between diabetes type 1 and type 2 and dementia subtypes below age 65 allowing more than one subtype per individual. 7](#_Toc10708877)

[Supplementary Table 5. Prospective associations between diabetes type 1 and type 2 and dementia during or after the first year after diabetes diagnosis. 8](#_Toc10708878)

[Supplementary Table 6. Baseline characteristics of the combined Glostrup cohorts. 9](#_Toc10708879)

[Supplementary Table 7. Baseline characteristics of the Addition study. 10](#_Toc10708880)

[Supplementary Table 8. Characteristics of the CAMB cohort. 11](#_Toc10708881)

[Supplementary Figure 1. Flow cart of participants in the national register study. 12](#_Toc10708882)

[Supplementary Figure 2. Prospective associations between diabetes type 1 and 2 and dementia above age 65 in the nationwide study allowing more than one subtype per individual. 13](#_Toc10708884)

# Supplementary text

### **Missing information in the cohorts**

In the combined Glostrup cohorts, 258 individuals without HbA1c measurement at baseline were excluded leaving 16,801 individuals for analysis. Of these, 144 (0.86%) individuals had missing information on education, 119 (0.71 %) on smoking status, 256 (1.52 %) on physical exercise, 12 (0.07 %) on body mass index and finally 1,814 (10.8 %) on alcohol use. As these missing data were assumed to be missing at random, all variables except for alcohol use were imputed based on age, sex and marital status. Alcohol use was categorized into 5 categories: a category for non-users, tertiles of alcohol use amount, and finally a group with missing information.

In the ADDITION study, 1,363 individuals without HbA1c measurement at baseline were excluded leaving 26,583 individuals for analysis. Of these 395 (1.5 %) had missing information on education and 762 (2.8 %) had missing information on physical activity. As missing data were assumed to be missing at random, they were imputed based on age, sex and marital status. For the remaining variables 2,349 (8.8%) had missing data on smoking status, and 2,049 (7.7 %) on body mass index. These variables were categorized, and missing information was placed in a separate category.

In the CAMB cohort, 5,535 individuals completed the IST2000R and had information on diabetes status. Of these, 5,408 (99%) had information on HbA1c. Of the 5,408, 20 (0.37 %) individuals had missing information on education, 24 (0.44 %) on whether they lived alone, 9 (0.17 %) on smoking status, 60 (1.11 %) on alcohol use, 29 (0.54 %) on bodyfat percentage, 32 (0.59 %) on high-sensitive CRP (hsCRP), 55 (1.02 %) on IL-6, 253 (4.68) on IL-10. Missing data was imputed based on age and sex.

# Supplementary Tables and Figures

### Supplementary Table 1. ICD10 and ATC codes used to define endpoints and covariables in the cohorts.

|  | |  |
| --- | --- | --- |
|  | **ICD10** | **ATC** |
| ***Endpoints*** |  |  |
| All dementia | G30-31, F00-03 |  |
| Alzheimer's dementia | G30, F00 |  |
| Vascular dementia | F01 |  |
| Other dementia | F02-03, G31 |  |
|  |  |  |
| ***Somatic comorbidity*** |  |  |
| Ischemic heart disease | I20-25 |  |
| Cerebrovascular disease | I60-69 |  |
| Cardiovascular medication |  | B01AC22, B01AC04,  B01AA03-04  B01AC06, N02BA01 |
| Hypertension | I10-12 | C03A  C07-08 |
| Obesity | E65-68 | A08 |
| Hypercholesterolemia | E780-782 | C10AA |
| Chronic obstructive pulmonary disease | J40-44 | R03 |
| Infection | A0-B9, J0-J2 L01-03, K35-37  N30, G0 | J01-05 |
| Inflammatory disease | M30-36, K50-54 D88, M05-15 L40 |  |
|  |  |  |
| ***Psychiatric comorbidity*** |  |  |
| Alcohol disorder | F10, G312, G621 G711, I426, K292 K70, K860, Z721 | N07BB |
| Major depression | F32-33 | N06A |
| Dementia medication* |  | N06D |
| ICD10=International Classification of Disease 10. ATC=Anatomical Therapeutic Chemical classification system codes. *Dementia medication was used to exclude individuals with previous dementia. | | |

| Supplementary Table 2. Exposure, outcomes, and covariables in the four cohorts. | | | | |
| --- | --- | --- | --- | --- |
|  | **Nationwide  study** | **Glostrup  Cohorts** | **Addition** | **CAMB** |
| Number | 290,588 | 16,780 | 26,536 | 5,408 |
|  |  |  |  |  |
| **Exposure** |  |  |  |  |
| Diabetes mellitus | NPR |  |  | Questionnaire |
| HbA1c ≥ 48 mmol/mol |  | Blood test | Blood test | Blood test |
|  |  |  |  |  |
| **Outcome** |  |  |  |  |
| All dementia | NPR | NPR | NPR |  |
| Alzheimer's dementia | NPR |  |  |  |
| Vascular dementia | NPR |  |  |  |
| Other dementia | NPR |  |  |  |
| Cognitive performance |  |  |  | IST2000R |
|  |  |  |  |  |
| **Covariables** |  |  |  |  |
| ***Basic variables*** |  |  |  |  |
| Age | CRS | Questionnaire | Questionnaire | Questionnaire |
| Sex | CRS | Questionnaire | Questionnaire | Questionnaire |
| Marrital status | CRS |  |  | Questionnaire |
| Education | IDA | IDA | IDA | Questionnaire |
|  |  |  |  |  |
| ***Illness variables*** |  |  |  |  |
| Ischemic heart disease | NPR |  |  |  |
| Cerebrovascular disease | NPR |  |  |  |
| Cardiovascular medication | DPR |  |  |  |
| Hypertension | NPR/DPR |  |  |  |
| Obesity | NPR/DPR |  |  |  |
| Hypercholesterolemia | NPR/DPR |  |  |  |
| Chronic obstructive pulmonary disease | NPR/DPR |  |  |  |
| Infection* | NPR/DPR |  |  |  |
| Inflammatory disease | NPR |  |  |  |
| Alcohol use disorder | NPR/DPR |  |  |  |
| Major depression | NPR/DPR | NPR/DPR | NPR/DPR |  |
|  |  |  |  |  |
| ***Clinical variables*** |  |  |  |  |
| Body mass index |  | Physical exam | Physical exam |  |
| Smoking status |  | Questionnaire | Questionnaire | Questionnaire |
| Alcohol use |  |  |  | Questionnaire |
| Physical exercise |  | Questionnaire | Questionnaire |  |
| Body fat |  |  |  | Physical exam |
| High sensitive C-reactive protein |  |  |  | Blood test |
| Interleukin 6 |  |  |  | Blood test |
| Interleukin 10 |  |  |  | Blood test |
| NDR = National Diabetes Register. CAMB = The Copenhagen Aging and Midlife Biobank. HbA1c = Glycosylated Hemoglobin, type A1C. IST2000R = Intelligenz-Struktur-Test 2000R. NPR=National Patient Register. CRS=the Civil Registration System. IDA=Integrated DAtabase for labor market research. DPR=the Danish Prescription Register. *Individuals with more than 10 prescriptions. | | | | |

| Supplementary Table 3. Prospective associations between diabetes type 1 and type 2 and dementia subtypes before age 65. | | | | |
| --- | --- | --- | --- | --- |
|  |  |  | HR (95% CI) | |
|  | N_total_ | N_endpoints_ | Adj_Age_ | Adj_Multi_ |
| **Age <65 years at dementia** | |  |  |  |
| All dementia |  |  |  |  |
| Reference | 87,646 | 344 | 1 [Reference] | 1 [Reference] |
| Diabetes type 1 | 13,719 | 88 | 2.75 (2.17-3.49) | 1.72 (1.35-2.20) |
| Diabetes type 2 | 73,731 | 609 | 2.04 (1.79-2.33) | 1.69 (1.47-1.95) |
| Alzheimers |  |  |  |  |
| Reference | 87,646 | 58 | 1 [Reference] | 1 [Reference] |
| Diabetes type 1 | 13,719 | MD | 1.33 (0.60-2.98) | 1.00 (0.44-2.26) |
| Diabetes type 2 | 73,731 | 63 | 1.25 (0.88-1.79) | 1.08 (0.72-1.60) |
| Vascular dementia |  |  |  |  |
| Reference | 87,646 | 13 | 1 [Reference] | 1 [Reference] |
| Diabetes type 1 | 13,719 | MD | 9.6 (4.20-22.0) | 4.44 (1.86-10.6) |
| Diabetes type 2 | 73,731 | 38 | 3.33 (1.78-6.26) | 2.07 (1.06-4.04) |
| Other dementia |  |  |  |  |
| Reference | 87,646 | 273 | 1 [Reference] | 1 [Reference] |
| Diabetes type 1 | 13,719 | 71 | 2.74 (2.10-3.57) | 1.70 (1.29-2.24) |
| Diabetes type 2 | 73,731 | 508 | 2.15 (1.86-2.49) | 1.80 (1.54-2.11) |
| HR=Hazard ratio, CI=Confidence Interval. Adjage=Adjusted for age, AdjMulti=multivariable adjusted. MD: categories with microdata, which we were not allowed to depict or numbers which would allow calculation of microdata. | | | | |
|  |  |  |  |  |

### Supplementary Table 4. Prospective associations between diabetes type 1 and type 2 and dementia subtypes below age 65 allowing more than one subtype per individual.

|  | | | | |
| --- | --- | --- | --- | --- |
|  |  |  | HR (95% CI) | |
|  | Ntotal | Nendpoints | AdjAge | AdjMulti |
| **Age <65 years at dementia** | |  |  |  |
| All dementia |  |  |  |  |
| Reference | 87,646 | 344 | 1 [Reference] | 1 [Reference] |
| Diabetes type 1 | 13,719 | 88 | 2.75 (2.17-3.49) | 1.72 (1.35-2.20) |
| Diabetes type 2 | 73,731 | 609 | 2.04 (1.79-2.33) | 1.69 (1.47-1.95) |
| Alzheimers |  |  |  |  |
| Reference | 87,646 | 67 | 1 [Reference] | 1 [Reference] |
| Diabetes type 1 | 13,719 | MD | 1.15 (0.52-2.55) | 0.83 (0.37-1.85) |
| Diabetes type 2 | 73,731 | 74 | 1.27 (0.92-1.78) | 1.08 (0.75-1.55) |
| Vascular dementia |  |  |  |  |
| Reference | 87,646 | 13 | 1 [Reference] | 1 [Reference] |
| Diabetes type 1 | 13,719 | MD | 10.6 (4.74-23.7) | 5.03 (2.17-11.7) |
| Diabetes type 2 | 73,731 | 43 | 3.78 (2.03-7.02) | 2.48 (1.29-4.76) |
| Other dementia |  |  |  |  |
| Reference | 87,646 | 296 | 1 [Reference] | 1 [Reference] |
| Diabetes type 1 | 13,719 | 74 | 2.66 (2.05-3.44) | 1.67 (1.28-2.18) |
| Diabetes type 2 | 73,731 | 526 | 2.05 (1.78-3.37) | 1.74 (1.49-2.03) |
| Based on 20,261 individuals with diabetes type 1 and 125,520 individuals with diabetes type 2. HR=Hazard ratio, CI=Confidence Interval. Adjage=Adjusted for age, AdjMulti=multivariable adjusted. MD: categories with microdata, which we were not allowed to depict. | | | | |

### Supplementary Table 5. Prospective associations between diabetes type 1 and type 2 and dementia during or after the first year after diabetes diagnosis.

| Supplementary Table 6. Baseline characteristics of the combined Glostrup cohorts. | | |
| --- | --- | --- |
|  | **HbA1c** | |
|  | **<48 mmol/mol** | **≥48 mmol/mol** |
| Number (n) | 16,142 (96) | 638 (4) |
| HbA1c in mmol/mol, mean (range) | 37 (33-40) | 53 (50-63) |
|  |  |  |
| ***Basic variables*** |  |  |
| Age in years, median (IQR) | 50 (40-57) | 55 (50-60) |
| Men, n (%) | 7,450 (46) | 413 (64) |
| Education, n (%) |  |  |
| Basic education | 3,167 (20) | 198 (31) |
| Medium education | 8,876 (55) | 336 (53) |
| Long education | 4,099 (25) | 104 (16) |
|  |  |  |
| ***Clinical variables*** |  |  |
| Smoking, n (%) |  |  |
| Never smoker | 6,823 (42) | 176 (28) |
| Previous smoker | 5,078 (32) | 201 (32) |
| Current smoker | 4,241 (26) | 261 (41) |
| Physical exercise, n (%) |  |  |
| Sedentary | 2,761 (17) | 181 (28) |
| Mild | 9,510 (59) | 380 (59) |
| Moderate | 3,677 (23) | MD* |
| Severe/hard | 215 (1) | MD* |
| Body mass index, median (IQR) | 25 (23-28) | 29 (26-33) |
|  |  |  |
| ***Illness variables*** |  |  |
| Major depression, n (%) | 254 (2) | 17 (3) |
| Based on 16,780 individuals in the combined Glostrup cohorts with a measurement of HbA1c. Individuals with a previous dementia is excluded (n=21). *MD=Microdata not allowed to report or data which allows calculation of microdata. HbA1c = Glycosylated Hemoglobin, type A1C. | | |

| Supplementary Table 7. Baseline characteristics of the Addition study. | | |
| --- | --- | --- |
|  | **HbA1c** | |
|  | **<48 mmol/mol** | **≥48 mmol/mol** |
| Number (n) | 25,747 (97) | 789 (3) |
| HbA1c in mmol, mean (range) | 37 (9-48) | 62 (49-142) |
|  |  |  |
| ***Basic variables*** |  |  |
| Age in years, mean (range) | 59 (36-72) | 59 (40-72) |
| Men, n (%) | 13,136 (51) | 457 (58) |
| Education, n (%) |  |  |
| Basic education | 8,473 (33) | 277 (35) |
| Medium education | 11,783 (46) | 392 (50) |
| Long education | 5,491 (21) | 120 (15) |
|  |  |  |
| ***Clinical variables*** |  |  |
| Smoking, n (%) |  |  |
| Never smoker | 8,356 (32) | 236 (30) |
| Previous smoker | 7,989 (31) | 251 (32) |
| Current smoker | 6,407 (25) | 265 (33) |
| Occationally smoker | 668 (3) | 23 (3) |
| Missing | 2,327 (9) | 14 (2) |
| Physical exercise |  |  |
| Active, n (%) | 4,810 (19) | 125 (16) |
| Body Mass Index, n (%) |  |  |
| <=25 | 6,365 (25) | 88 (11) |
| >25-30 | 10,960 (43) | 240 (30) |
| >30-35 | 4,801 (19) | 279 (35) |
| >35 | 1,589 (6) | 173 (22) |
| Missing | 2,032 (8) | <10* |
|  |  |  |
| ***Illness variables*** |  |  |
| Major depression diagnosis, n (%) | 371 (1) | 18 (2) |
| Individuals with a previous dementia is excluded (n=50) leaving 26,536 individuals for analysis. HbA1c = Glycosylated Hemoglobin, type A1C. *Microdata, not allowed to report. | | |

### Supplementary Table 8. Characteristics of the CAMB cohort.

|  | | |
| --- | --- | --- |
|  | **No diabetes** | **Diabetes** |
| Number (n) | 5,169 (96) | 239 (4) |
| HbA1c, mean (range) | 34 (5-48) | 49 (24-105) |
|  |  |  |
| ***Basic variable*** |  |  |
| Age in years, mean (range) | 54 (49-62) | 56 (49-62) |
| Men, n (%) | 3,517 (68) | 195 (82) |
| Education, n (%) |  |  |
| Basic education | 566 (11) | 37 (15) |
| Skilled worker | 1,826 (35) | 100 (42) |
| Short to medium education | 1,862 (36) | 80 (33) |
| Long education | 875 (17) | 21 (9) |
| Other education | 40 (1) | 1 (0) |
| Living alone, n (%) | 763 (15) | 64 (27) |
|  |  |  |
| ***Clinical variables*** |  |  |
| Alcohol use (units/week), mean (range) | 12 (0-160) | 14 (0-133) |
| Smoking, n (%) |  |  |
| Never smoker | 1,880 (36) | 70 (29) |
| Previous smoker | 2,102 (41) | 34 (45) |
| Periodic smoker | 138 (3) | 1 (1) |
| Daily smoker | 1,042 (20) | 19 (25) |
| Bodyfat (%), mean (range) | 24 (3-50) | 27 (5-51) |
| High sensitive C-reactive protein, mean (range) | 2 (0-108) | 4 (0-47) |
| Interleukin-6, mean (range) | 4 (0-442) | 5 (0-80) |
| Interleukin-10, mean (range) | 12 (0-7,352) | 20 (0-2351) |
| Based on 5,408 individuals in the Copenhagen Aging and Midlife Biobank (CAMB). Diabetes was defined as either Hba1c ≥48 or self-reported diabetes. HbA1c=Glycosylated Hemoglobin, type A1C. | | |

### Supplementary Figure 1. Flow cart of participants in the national register study.

###
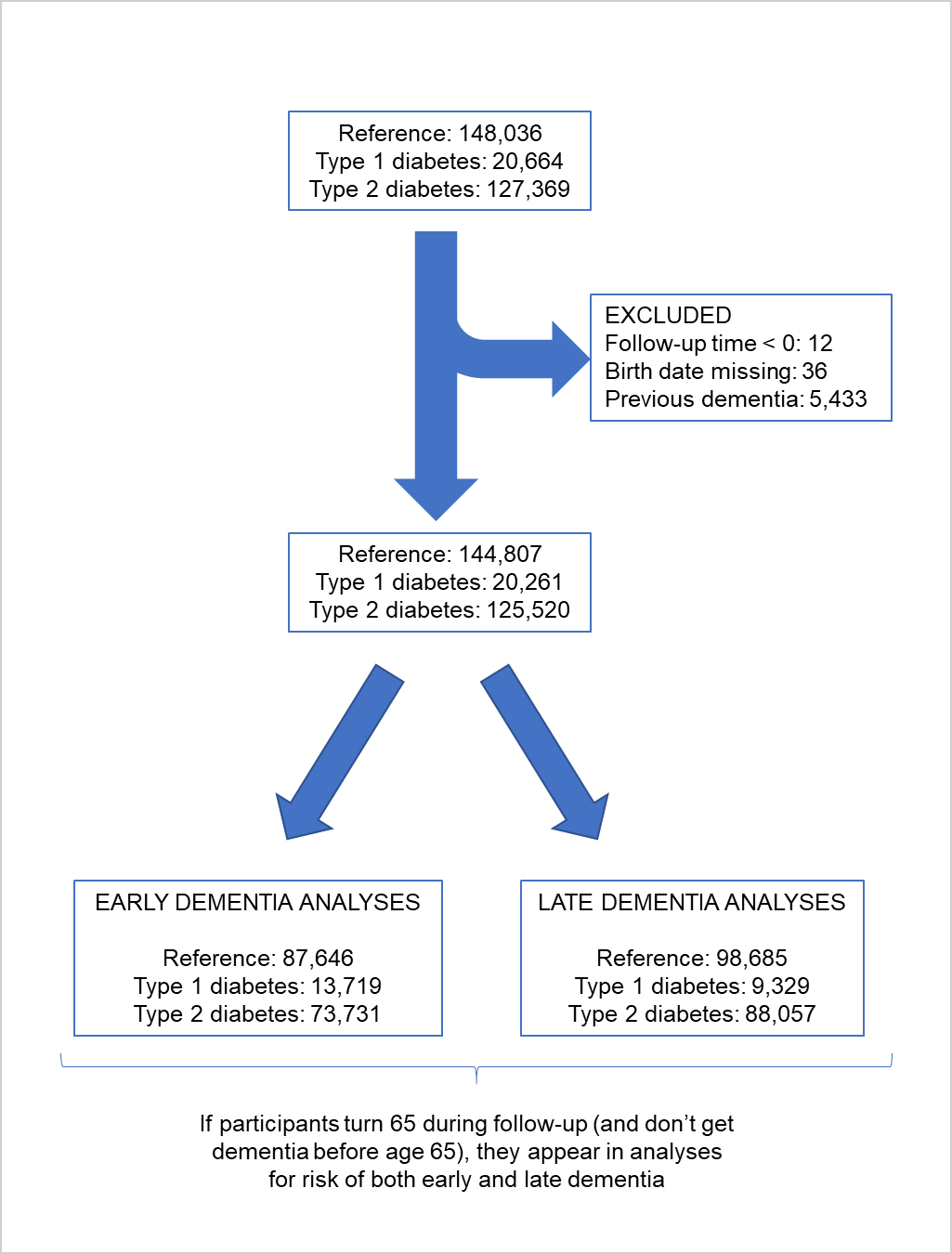


### Supplementary Figure 2. Prospective associations between diabetes type 1 and 2 and dementia above age 65 in the nationwide study allowing more than one subtype per individual.


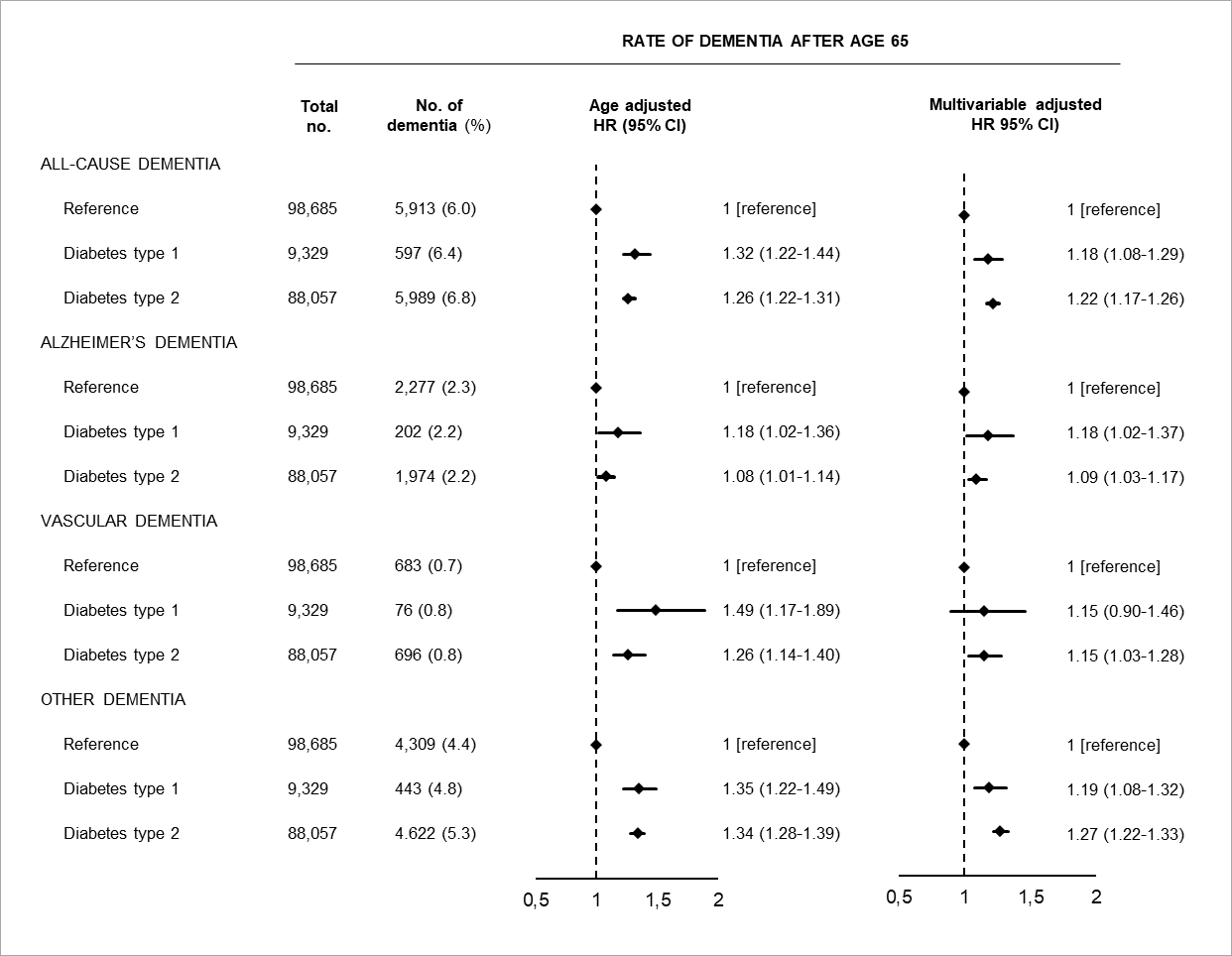


Multivariable adjustment was for age, sex, marital status, education, register-based ischemic heart disease, cerebrovascular disease, vascular disease medication, hypertension, obesity, hypercholesterolemia, infections, chronic obstructive pulmonary disease, inflammatory diseases, depression and alcohol use disorders. HR = hazard ratio. CI = confidence interval. In these analyses (contrary to in Figure 1) we allowed individuals to have more than one dementia subtype, and consequently the total number of all-cause dementia may be lower than the subtypes combined.
